# GNN-RMNet: Leveraging graph neural networks and GPS analytics for driver behavior and route optimization in logistics

**DOI:** 10.1371/journal.pone.0328899

**Published:** 2025-08-07

**Authors:** Eman Ali Aldhahri, Abdulwahab Ali Almazroi, Monagi Hassan Alkinani, Mohammed Alqarni, Elham Abdullah Alghamdi, Nasir Ayub

**Affiliations:** 1 Computer Science and Artificial Intelligence Department, Collage of Computer Science and Engineering, University of Jeddah, Jeddah, Saudi Arabia; 2 College of Computing and Information Technology at Khulais, Department of Information Technology, University of Jeddah, Jeddah, Saudi Arabia; 3 Department of Software Engineering, College of Computer Science and Engineering, University of Jeddah, Jeddah, Saudi Arabia; 4 Department of Creative Technologies, Faculty of Computing and Artificial Intelligence, Air University Islamabad, Islamabad, Pakistan; Prince Sultan University, SAUDI ARABIA

## Abstract

Logistics networks are becoming increasingly complex and rely more heavily on real-time vehicle data, necessitating intelligent systems to monitor driver behavior and identify route anomalies. Traditional techniques struggle to capture the dynamic spatiotemporal relationships that define driver actions, route deviations, and operational inefficiencies in big fleets. This paper introduces GNN-RMNet, a hybrid deep learning system that combines GNN, ResNet, and MobileNet for interpretable, scalable, and efficient driver behavior profiling and route anomaly detection. GNN-RMNet utilizes spatiotemporal GPS trajectories and vehicle sensor streams to learn contextual and relational patterns from structured driving data in real time, thereby identifying dangerous driving and route violations. On a real-world GPS-vehicle sensor dataset, the proposed model achieves 98% accuracy, 97% recall, an F1-score of 97.5%, and domain-specific measures like Anomaly Detection Precision (96%) and Route Deviation Sensitivity (95%). Modular design offloads ResNet-GNN analytics to edge nodes while preserving MobileNet components for on-vehicle inference, resulting in reduced inference latency (32 ms). Comparing GNN-RMNet against baseline, ensemble, and hybrid models shows its accuracy, efficiency, and generalization advantages. Computational feasibility, anomaly scoring interpretability, and future deployment concerns, including cybersecurity, data privacy, and multimodal sensor integration, are all covered. For real-time fleet safety management and secure, intelligent, and context-aware logistics, GNN-RMNet seems promising. The framework incorporates multimodal, privacy-aware, and scalable driver analytics, enabling its use in intelligent transportation systems and urban logistics infrastructures.

## Introduction

The digital revolution of logistics is changing business models, processes, and technology [1]. The integration of modern technologies, such as deep learning and GPS-based analytics, has improved operational efficiency and road safety as transportation ecosystems continue to grow. Road traffic safety is vital in this growth since traffic accidents may cost a lot and kill people. Accident causes include traffic congestion, reckless driving, poor vehicle control, and bad environmental conditions [2, 3]. Driver behaviour is the main risk element, hence predictive analytics is essential for risk assessment and reduction. Recently, autonomous driving and ADAS have been studied for real-time behavioral monitoring and anomaly identification. Multiple sensor and AI algorithms identify and prevent dangerous driving. Studies have identified speed changes and engine dynamics as potential problems using On-Board Diagnostics (OBD) interfaces [4]. Vision-based systems and inertial sensors like cameras and gyroscopes are used to detect driver intent and vehicle instability [5]. Simulations may simulate abnormal driving behaviors in controlled, dynamic circumstances [6]. Highway surveillance systems may capture vehicle trajectory data for real-time examination of anomalous driving [7]. Recognition of unsafe driving behaviors is difficult, particularly in imbalanced datasets with low critical event frequencies, despite technical progress. C-ITS will improve driving behavior analysis with seamless V2X communication. By simplifying data exchange, C-ITS enhances traffic efficiency, safety, and comfort, including CAMs and DENMs [8]. Despite its potential, C-ITS impacts on driver behavior and road safety remain unknown. Since C-ITS is cooperative, it creates a lot of data, which may help monitor system performance and detect safety concerns early. Vehicle-to-Infrastructure (V2I) communications rely on Road-Side Units (RSUs) in poor C-ITS vehicle coverage regions [9]. Advanced functionalities like TLM, RLT, and IVI provided by RSUs improve routing and driver decision-making [10]. System monitoring and anomaly detection are essential since RSU infrastructure failures may significantly restrict these capabilities. CAN bus signals included in modern driver behavior profiles provide real-time insight into vehicle dynamics and operational behavior [11]. Data anonymization and secure multiparty computing are becoming more common as systems integrate biometric and vehicular data [12]. In connected car ecosystems, edge-based distributed analytics frameworks are preferred due to their low latency and reduced dependence on centralized infrastructure [13]. These trends indicate that intelligent logistics networks will use context-aware, resilient, and ethical driver behavior analytics.

Technology has made progress, but cybersecurity weaknesses remain. Previous research has identified weaknesses in authentication and encryption techniques, even in safety-critical areas such as marine and transit navigation [14]. These gaps pose a threat to driver safety and operational continuity. Secure communication, real-time monitoring, and intelligent anomaly detection systems are needed to address these issues. A deep learning-driven system that uses GPS trajectory analytics to predict driver behavior and identify logistics fleet route abnormalities is presented in this paper. Advanced preprocessing, MRMNS, and Enhanced Orthogonal Projection Transformation are used in the suggested strategy. Together, these components increase model interpretability and detection. This study provides logistics operators and safety stakeholders with practical insights for proactive risk management and data-driven decision-making in smart transportation networks.

1. Introduction of Risk-Aware Behavioral Characteristics Introduced to capture complex elements of unsafe driving behavior were practical indicators like Risk-Adjusted Speed (RAS), Acceleration Variability (AV), Stop Intensity Index (SII), Fuel Efficiency Ratio (FER), and Route Deviation Severity (RDS).
2. Enhanced Feature Selection Using MRMNS: The Maximum Relevance–Minimum Noise Selection (MRMNS) technique was used to underline pertinent features while so suppressing noisy inputs, thus improving the interpretability and stability of the model.
3. Correcting Class Imbalance for Rare Anomalies: To increase detection accuracy for minority classes—especially those connected to crucial but rare driving anomalies—a new data-balancing method was used.
4. Graph neural networks, ResNet, and MobileNet were combined to provide a new categorization framework, GNN-RMNet, in hybrid deep learning architecture. For efficient and effective anomaly detection, this architecture leverages relational, spatial, and lightweight computing capabilities.
5. Creation of Domain-specific Assessment Measures: Formulated two specific metrics—Anomaly Detection Precision (ADP) and Route Deviation Sensitivity (RDS)—to evaluate the model’s performance in the framework of behavioral analytics and logistics safety.
6. Extensive studies revealed that the suggested approach offers up to 15% more accuracy, 12% improvement in precision, and 10% increase in recall when compared with current methods, so stressing its possible influence on intelligent transportation systems and fleet management.

The article begins by examining recent developments in artificial intelligence, with a particular emphasis on its applications within the domain of transportation analytics. Building upon this foundation, it introduces a novel conceptual framework that leverages a GNN-RMNet ensemble for modeling driver behavior and detecting route anomalies. This is followed by an in-depth presentation and analysis of the experimental data, highlighting the performance and implications of the proposed method. The article concludes by synthesizing the key findings and outlining potential avenues for future research.

## Related work

Due to GPS data and deep learning algorithms, driver behavior analysis and route anomaly detection are becoming more crucial in logistics. Recent advances in driver behavior modeling also explore personalized representations for interactive systems [15, 16]. The author [17] utilized a deep learning framework to identify abnormalities in logistics routes based on driver behavior. This approach found route inefficiencies and unusual deviations using previously collected GPS data and behavioral models. This approach may overlook real-time traffic patterns. Real-time monitoring systems may reduce dangers associated with driver fatigue and distractions, which can impact road logistics [18]. Focusing solely on GPS data may overlook behavioral factors that contribute to route deviations.

Route anomalies may occur in human-driven and autonomous vehicles due to atypical driving behavior or road conditions. Author[19, 20] used GPS trajectory clustering to identify anomalous delivery route patterns. The analysis revealed significant deviations from planned routes; however, the limited ability of clustering algorithms to adapt to traffic events may not accurately reflect real-world issues. In logistics operations, [21] used machine learning-based driver behavior analysis to predict route anomalies. Their work focuses on combining behavioral data with GPS analytics. However, it does not provide adaptation to the real-time model. Understanding the short-term lateral behavior of vehicles is vital for modeling driver intentions and anomaly detection, as shown in [22].

According to [23], an IoT-based logistics monitoring system utilizes GPS data and sensor analytics to detect driving behaviors and identify vehicle issues. Their system optimizes routes but does not account for unexpected road closures. Research suggests that combining IoT devices with deep learning algorithms may enhance the efficiency of driver behavior monitoring [24]. Spatiotemporal graph transformers have enhanced traffic prediction frameworks [25], aligning with our GNN-based structure. [26] advocates employing neural network models to recognize driver distractions and decrease human errors. This strategy enhances anomaly identification but requires the integration of multiple data sources, including weather data and road conditions. Trajectory planning under uncertainty has been explored using risk-aware models [27], which informs the development of safer logistics routing strategies.

Predicting vehicle movement based on driving styles has proven effective for preempting abnormal behavior [28]. In [29], GPS data and deep learning models were used to detect route abnormalities in real time, using a Raspberry Pi framework for route monitoring. This innovative technology might detect logistics route abnormalities. However, GPS signal accuracy can be compromised in congested metropolitan areas. In addition, [30] developed a machine learning-based approach to detect fatigue in long-haul drivers. Their weariness detection method is effective for long-distance circumstances but may not be suitable for short-haul logistics. [31] suggests that adaptive learning models can manage dynamic route changes while rule-based systems can detect odd driving patterns. These models may need to respond to rapidly changing traffic conditions, which can reduce their long-term efficacy. Researchers [32] used GPS data and deep learning to identify route irregularities in logistics networks. While sophisticated analytics are crucial, their research does not fully address real-time behavioral features, making route anomaly detection prediction models insufficient.

Integrating GPS data analytics and deep learning ensures the effective identification of logistical anomalies. The research proposes that integrating traffic and behavioral data might assist in detecting logistical route abnormalities [33]. Their results are exciting, but real-time data processing constraints may limit their use. The Multilayer Neural Network Model [34] integrates data from several sources to provide a comprehensive route anomaly detection system. Using this paradigm may be computationally and resource-costly. A study by [35] identified a link between aggressive driving, route deviations, and fuel inefficiency. However, GPS data alone may fail to capture driving behavior patterns in real-time route selection. Emotion-aware multimodal learning has shown promise in improving behavioral prediction accuracy [36].

According to [37], decision trees can analyze route anomalies and discover behaviors that depart from expected logistical paths. However, their reliance on historical data may lead to inaccurate future estimates. By learning from dynamic traffic scenarios, Deep learning algorithms enhance logistics monitoring and warning systems. In [38], a predictive route optimization system was created to improve delivery times by monitoring driver behavior. This strategy enhances delivery routes but needs to address traffic or road closure difficulties. Using IoT devices and GPS analytics, the author [39] observed real-time driving behavior in logistics operations and gave alerts for immediate action. Although promising, this strategy is only suitable for urban logistics and does not handle long-distance or rural logistics issues. Table 1 shows the summarized view of the literature.

**Table 1:** Literature summarized view.

Ref	Technique Used	Objective Achieved	Limitations
[17]	Recurrent Neural Networks (RNN) for driver behavior-based anomaly detection	Detected route inefficiencies and unusual deviations	Overlooks real-time traffic patterns
[18]	Real-time monitoring systems using sensor fusion	Reduced dangers related to driver fatigue and distractions	May ignore other behavioral factors contributing to route deviations
[19]	K-means clustering for GPS trajectory anomaly detection	Identified significant route deviations from planned paths	Limited adaptability to real-time traffic events
[20]	DBSCAN clustering for identifying delivery route deviations	Revealed route deviations and inefficiencies in logistics	Clustering algorithms may not fully adapt to dynamic traffic conditions
[21]	Support Vector Machine (SVM) for driver behavior-based anomaly prediction	Predicted route anomalies using behavioral and GPS data	Does not adapt well to real-time models
[23]	IoT-based monitoring with GPS and sensor fusion	Optimized routes by identifying driving behaviors and vehicle issues	Does not account for unexpected road closures
[24]	Convolutional Neural Networks (CNN) for driver behavior monitoring	Improved driver behavior monitoring	Requires integration of multiple data sources (e.g., weather conditions)
[26]	Feedforward Neural Networks for detecting driver distractions	Reduced human errors by identifying distractions	Requires data source integration (e.g., weather, road conditions)
[29]	Long Short-Term Memory (LSTM) for real-time route anomaly detection	Detected route abnormalities in real-time logistics routes	GPS signal accuracy compromised in urban areas
[30]	Random Forest for driver fatigue detection in long-haul drivers	Detected fatigue in long-haul drivers	Effective for long-haul drivers, not short-haul operations
[31]	Adaptive boosting (AdaBoost) for dynamic route change detection	Managed dynamic route changes and odd driving patterns	May struggle to adapt to rapidly changing traffic conditions
[32]	Multilayer Perceptron (MLP) for route irregularity detection	Identified route irregularities using GPS data and deep learning	Does not address real-time behavioral factors
[33]	Ensemble methods combining GPS and behavioral data	Assisted in detecting logistical route abnormalities	Real-time data processing limitations
[34]	Multilayer Neural Network for data fusion	Provided comprehensive route anomaly detection	Computationally and resource-costly
[35]	Regression analysis for linking aggressive driving to fuel inefficiency	Linked aggressive driving to fuel inefficiency and route deviations	Relies heavily on GPS data, may not capture real-time behaviors
[37]	Decision Tree for route anomaly classification	Analyzed route anomalies and identified deviations from logistical paths	Dependent on historical data, leading to erroneous estimates
[38]	Genetic Algorithms for predictive route optimization	Improved delivery times through route optimization	Needs to address traffic or road closure issues
[39]	IoT-based monitoring with GPS and decision tree alerts	Monitored real-time driving behavior in urban logistics	Limited to urban logistics, not effective for long-distance scenarios

### Limitations and gaps in existing research

Current research emphasizes many cybersecurity strategies in maritime navigation and logistics systems. Yet, a significant need persists in the area of driver behavior and route anomaly detection in logistics. A substantial portion of the study focuses on identifying abnormalities in navigation systems, including AIS and NMEA protocols. Moreover, there is a need for studies that explore real-time monitoring and predictive analytics of driver behavior using GPS data and deep learning algorithms.

Research on anomaly detection mainly focuses on preset rule-based systems or machine learning models developed from previous data. These solutions are limited by their lack of dynamic adaptability to shifting traffic conditions, driver behavior, or emerging danger situations. This is especially vital in logistics, where factors often remain unexpected. Moreover, most research on GPS data focuses on vehicle-level analysis rather than examining the intricacies of driver behavior that may lead to route deviations or safety hazards.

Deep-learning logistics methods are often limited by GPS data quality and granularity. Effective anomaly detection systems may be hindered by GPS signal accuracy fluctuations, especially in urban or wet weather. Additionally, minimal research has linked driver behavior analytics with GPS data to give a holistic view on logistical operations, making it difficult to understand how driver behaviors affect route efficiency and safety. Finally, although deep learning models have promise in predictive analytics, their real-time usage in logistics, particularly for route irregularities, requires additional study. Mixing deep learning models with real-time GPS data to discover driver-induced route deviations is the study’s solution. Logistics operations will be more thorough and adaptable.

## Proposed methodology

**Fig 1:**
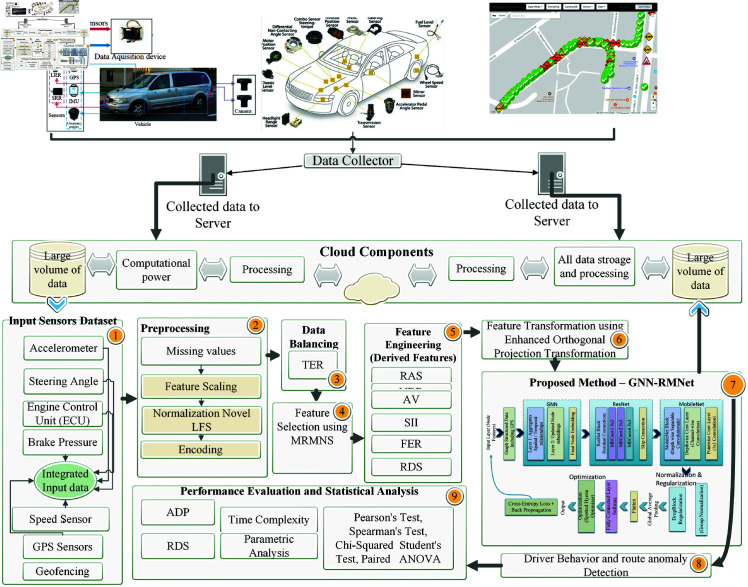
Proposed framework.

This study uses deep learning and GPS data analytics to analyze logistics driver behavior and detect route anomalies. The method uses GNN-RMNet architecture. This design uses GNNs for relational data processing, ResNet for deep feature extraction, and MobileNet for calculation speedup. Data is extensively prepared to accommodate missing values, normalize sensor readings, and address class imbalances via targeted event resampling. Intelligent features that capture driving patterns are created using feature engineering and the Maximum Relevance-Minimum Noise Selection (MRMNS) method. The Enhanced Orthogonal Projection Transformation (EOPT) optimizes the specified attributes to prepare for classification. The GNN-RMNet model aggregates graph-structured input utilizing GNN layers, ResNet’s residual connections, and MobileNet’s depthwise separable convolutions to achieve high accuracy and low computational costs. Performance is assessed by accuracy, precision, recall, F1-score, and newly introduced metrics, such as Anomaly Detection Precision (ADP) and Route Deviation Sensitivity. The approach has improved driver behavior analysis and logistics anomaly spotting in extensive simulations. Fig 1 shows this framework’s components and is explained in depth in future sections.

### Dataset description

This research uses deep learning and GPS data analytics to monitor logistics driver behavior and discover route anomalies. It includes auto sensor data of regular and abnormal driving behaviors such as abrupt braking, fast acceleration, unexpected lane changes, and route deviations. Vehicle sensors, such as GPS, accelerometers, gyroscopes, and ambient environment sensors, gather data. The sensors track vehicle performance and driver interactions during the journey. Sensor data was rigorously preprocessed to guarantee data integrity. Only sensor streams with confirmed calibration information were retained to minimize hardware inconsistencies. Temporal smoothing and Kalman filter-based interpolation enhanced GPS signal accuracy in urban environments with multipath effects and obstructions. This decreased signal noise and position drift, particularly in busy cities. Missing values were fewer than 3.5% of the dataset. The mean values were used to impute numerical information, whereas model-based imputation was used for qualitative features like weather and road type. Corrupt or missing sensor data were removed to avoid model training bias or instability. Note that the information covers urban, suburban, and highway driving conditions in a single location. This offers a realistic basis for simulating intra-city logistical activities, but its geographic and demographic breadth is restricted. One regional logistics network provides most of the data, and participating drivers are a demographic category associated with that operator. We recognize this constraint and aim to gather data from additional geographic regions and demographically diverse fleets to enhance generalizability. Intelligent transportation systems, road safety, and driver behavior monitoring studies benefit from the dataset’s anomaly-detection model training in real-world logistical settings. The dataset is available on an open repository at [40], with a thorough explanation in Table 2.

**Table 2:** Dataset features and descriptions.

S.No	Feature	Description
1	Trip ID	Unique identifier for each trip taken by the driver.
2	Driver ID	Unique identifier for the driver.
3	Vehicle ID	Unique identifier for the vehicle being driven.
4	Timestamp	Date and time when the GPS reading was recorded.
5	Latitude	GPS latitude coordinate for tracking vehicle location.
6	Longitude	GPS longitude coordinate for tracking vehicle location.
7	Speed	Speed of the vehicle at a given time.
8	Acceleration / Deceleration	Rate of speed change to identify sudden acceleration or braking events.
9	Steering Angle	Angle of the steering wheel to track swerving or sharp turns.
10	Heading	The direction the vehicle moves is measured in degrees (0^°^ to 360^°^).
11	Trip Duration	Total trip time from start to finish.
12	Trip Distance	Total distance traveled during the trip.
13	Fuel Consumption	Amount of fuel used during the trip.
14	RPM (Revolutions Per Minute)	Engine revolutions per minute during the trip.
15	Brake Usage	Frequency and intensity of brake usage during the trip.
16	Lane Deviation	Measure whether the vehicle deviates from its lane.
17	WeatherWeatherweather Conditions	Environmental conditions (e.g., sunny, rainy) during the trip.
18	Road Type	Type of road being driven on (e.g., highway, urban, rural).
19	Traffic Condition	Traffic density and flow conditions during the trip.
20	Stop Events	Number of stops made during the trip.
21	Geofencing Violation	Indicator of whether a geofence was crossed during the trip.
22	Anomalous Event	Indicates if any anomalous driving behavior occurred (e.g., harsh braking).
23	Route Anomaly	Indicates if the vehicle deviated from the planned route or geofencing rules.

### Preprocessing step

Preprocessing is crucial for preparing the dataset for machine learning models. This includes missing values, scaling features, encoding categorical variables, and class imbalance. Numerical variables like speed, acceleration, and fuel consumption are imputed using the mean, while categorical variables like weather and road type use the most common value to address missing values. An imputation method for missing values *M*_*p*,*q*_ is defined as [41]:

$$M_{p,q}=\left\{ \begin{array}{cc} \text{mean}(M_{q}) & \text{if }M_{p,q}\text{ is numerical}\\ \text{mode}(M_{q}) & \text{if }M_{p,q}\text{ is categorical} \end{array}\right.$$ (1)

To assure similarity, min-max normalization scales continuous data, including speed, acceleration, RPM, and trip length. This is crucial for distance-based algorithms. Normalization is done as [41]:

$$N_{\text{scaled}}=\frac{N-\min(N)}{\max(N)-\min(N)}$$ (2)

We employ one-hot encoding to translate category data like WeatherWeatherweather, road type, and traffic situation into binary vectors for machine learning models:

$$\text{One-hot}(C_{\text{cat}})=[0,1,0,\ldots ]$$ (3)

Class imbalance is present in this dataset, particularly due to the limited number of anomalous occurrences and route abnormalities. To handle this, we provide a new technique called Targeted Event Resampling (TER). TER oversamples minority classes according to their relevance and probability, unlike SMOTE, which generates synthetic samples equally. Integrating multi-source data streams for real-time decision-making is critical, as exemplified by urban transit systems like Du-Bus [42]. Oversampling rarer and more severe anomalies helps the model learn from these crucial occurrences more aggressively. The TER approach is:

$$\text{TER}(R)=\frac{\text{Minority Class Size}\times W_{\text{importance}}}{\text{Majority Class Size}}\times \text{Selective Synthetic Data}$$ (4)

The weight factor $W_{\text{importance}}$ modifies the number of synthetic samples dependent on the severity and relevance of the minority class. This tailored technique for class imbalance balances the dataset without oversampling less essential anomalies, keeping the emphasis on the most important occurrences. Thus, preprocessing ensures data cleansing, scalability, and the management of abnormal event identification and data imbalance, thereby preparing the dataset for modeling.

### Feature engineering

Feature engineering involves creating new, useful features from raw data, thereby improving machine learning models. We aim to identify new characteristics that more accurately capture driver behavior, environmental conditions, and route anomalies in this dataset. Extracting significant relationships between original features enhances the model’s anomaly detection and classification capabilities. Five additional data-derived characteristics follow. Speed is crucial in identifying unusual driving behavior, but the type of road also affects it. Highway speeding is less uncommon than urban speeding. Risk-adjusted speed (RAS) adjusts vehicle speed for road type. Calculation for this feature:

$$RAS=\frac{S}{R_{\text{type}}}$$ (5)

In this context, *S* denotes the speed of the vehicle, and $R_{\text{type}}$ stands for the risk factor associated with the kind of road (for instance, highways have lesser risk and urban roads are tremendous). Acceleration Variability (AV) and sudden acceleration or deceleration indicate aggressive driving. To measure acceleration variability throughout the journey, we compute the Acceleration Variability (AV). This may help spot extreme driving habits. Computation of acceleration standard deviation yields the characteristic:

AV=σ(A) (6)

σ(A) denotes the standard deviation of the trip acceleration numbers, and *A* stands for trip acceleration numbers. The third statistic is the Stop Intensity Index (SII). Stopping and starting during a drive may suggest impaired traffic navigation. We created the Stop Intensity Index (SII) to assess stop intensity based on trip duration and number of stops.

$$SII=\frac{N_{\text{stops}}}{T_{\text{trip}}}$$ (7)

The number of stops is denoted by $N_{\text{stops}}$, and the total duration of the journey is denoted by $T_{\text{trip}}$. Higher SII numbers indicate more stops, potentially owing to traffic or uncertain driving. FER, the fourth measure, impacts driving behavior and vehicle performance. The Fuel Efficiency Ratio (FER) calculates a driver’s fuel efficiency based on distance. Calculation for this feature:

$$FER=\frac{D_{\text{trip}}}{C_{\text{fuel}}}$$ (8)

The entire journey distance is denoted by $D_{\text{trip}}$ and fuel consumption is denoted by $C_{\text{fuel}}$. Lower *FER* scores may indicate inefficient driving, such as excessive acceleration or pauses. Route Deviation Severity (RDS) is the sixth statistic. Deviations from planned routes, especially in high-risk areas or traffic, may indicate anomalous behavior. The RDS feature controls deviations based on traffic and weather risks.

$$RDS=D_{\text{route}}\times (\text{Traffic Risk}+\text{Weather Risk})$$ (9)

The variables $D_{\text{route}}$ and Traffic Risk are used to describe different types of traffic, such as higher traffic and rainy or foggy weather, respectively. In several contexts, this function serves to make the gravity of route deviations more apparent.

### Feature selection via MRMNS

Features selected must be related to the aim variable and not cause model noise or instability. Our new feature selection method, MRMNS, tackles this problem. This method identifies useful traits and filters out noise to provide a reliable subset. Relevance and noise metrics evaluate each characteristic and establish selection criteria. Features’ relevance is evaluated using a modified mutual information measure, Normalized Relevance (NR). Targeting variable entropy balances relevance and information to normalize mutual information. We compute normalized relevance for feature *F*_*i*_ and target variable *Y*:

$$NR(F_{i},Y)=\frac{MI(F_{i},Y)}{H(Y)}$$ (10)

the entropy of the target variable *Y*, which quantifies uncertainty, and the mutual information between the feature *F*_*i*_ and the target variable *Y* are denoted as *MI*(*F*_*i*_,*Y*). Normalizing mutual information prevents large value ranges from dominating the selection process. To measure feature noise, we propose Feature Noise (FN). Noise is quantified by the feature’s variance conditioned on the target variable, which measures feature value fluctuations compared to target classes. Noise for feature *F*_*i*_ is computed as:

$$FN(F_{i})=\frac{1}{n}\sum_{k=1}^{n}\sigma (F_{i}|Y_{k})$$ (11)

Where $\sigma (F_{i}|Y_{k})$ is the conditional variance of the feature *F*_*i*_ given that the target variable *Y* takes the value *Y*_*k*_, and *n* is the number of distinct values *Y* can take. A high noise value indicates that the feature behaves inconsistently across different target classes, suggesting it may introduce instability into the model.

**Maximum relevance-minimum noise criterion:** A selection criteria that balances feature significance and noise underpins MRMNS. The selection score *S*(*F*_*i*_) for each feature is:

$$S(F_{i})=NR(F_{i},Y)-\alpha FN(F_{i})$$ (12)

The value of *NR*(*F*_*i*_,*Y*) represents the normalized relevance of the feature, *FN*(*F*_*i*_) represents the feature noise, and *α* determines the balance between relevance and noise. The parameter *α* is continuously modified to account for dataset noise. Higher *α* values penalize noisy features, guaranteeing a stable and meaningful subset. The MRMNS technique iteratively selects the most informative and least noisy features. Here’s an automated summary:

**Algorithm 1. Maximum Relevance-Minimum Noise Selection (MRMNS).**

1: Initialize an empty set S of selected features.

2: **for** each feature *F*_*i*_ in the dataset **do**

3:   Calculate normalized relevance *NR*(*F*_*i*_,*Y*).

4:   Compute feature noise *FN*(*F*_*i*_).

5:   Compute the selection score $S(F_{i})=NR(F_{i},Y)-\alpha FN(F_{i})$.

6: **end for**

7: Select the feature *F*_*i*_ with the highest selection score *S*(*F*_*i*_).

8: Add the selected feature *F*_*i*_ to the set S.

9: Repeat steps 2-6 until the desired number of features is selected or the selection scores converge.

The system scores features based on relevance and noise, and the feature with the highest selection score is added to *S*. Iteratively updating selection scores for remaining characteristics until the appropriate number is picked. The MRMNS approach requires adaptive modification of the parameter *α* to regulate the weight of the noise component in the selection criteria. If the dataset has considerable noise, boost *α* to favor stable features. To emphasize relevance, lower *α* if the dataset is pretty clean. This adaptable mechanism makes the technique versatile and can handle varying levels of data noise.

### Feature transformation via enhanced orthogonal projection transformation

After feature selection, feature transformation must optimize feature representation for classification. We introduce EOPT, a novel approach that translates selected characteristics into an orthogonal space, maximizing separation while preserving variance and reducing correlation. EOPT transforms features into highly informative and independent ones by utilizing orthogonal projection and variance maximization, thereby improving classification performance. EOPT uses orthogonal projection to transfer specified characteristics $X_{1},X_{2},\ldots ,X_{n}$ into a mutually orthogonal space. The resulting feature set is linearly independent after this modification, reducing redundancy and multicollinearity. Definition of the orthogonal projection matrix *P* for a feature *X* onto a vector *v* [43]:

$$P(X)=\frac{X\cdot v}{v\cdot v}v$$ (13)

The orthogonal vector in the new space is *v*. To create orthogonal, independent features, each chosen feature is projected onto its orthogonal basis. Although orthogonal projection lowers duplication, it may minimize variance, which is essential for data preservation. The variance maximizing phase in EOPT addresses this. The strategy maximizes the variance of transformed features after projecting them to preserve as much information as possible. A modified feature *T* has a variance $\sigma^{2}(T)$:

$$\sigma^{2}(T)=\frac{1}{n}\sum_{i=1}^{n}(T_{i}-\mu_{T}{)}^{2}$$ (14)

*T*_*i*_ represents the modified feature values, $\mu_{T}$ represents the mean of *T*, and *n* represents the sample count. This maintains a high variance in converted features, preserving crucial data patterns.

By optimizing orthogonality and variance, EOPT balances these two factors. Maximizing the variance of the changed features while minimizing correlation determines the transformation matrix *W*. The EOPT objective function is:

$$W^{*}=\arg\max_{W}\left(\sum_{i=1}^{n}\sigma^{2}(T_{i})-\beta \sum_{i\neq j}\text{Corr}(T_{i},T_{j})\right)$$ (15)

This approach, as outlined in Algorithm 2, initially projects the chosen characteristics onto an orthogonal basis to ensure linear independence. The next stage is to maximize the variance of converted features and minimize correlation. The feature set is optimized for classification after this approach. The powerful EOPT technique transforms features to be orthogonal and keeps large variance. This makes features informative and suitable for categorization, thereby improving the performance of machine learning models. EOPT optimizes the feature space via orthogonality and variance maximization, thereby preparing the data for the machine learning process.

**Algorithm 2. Enhanced Orthogonal Projection Transformation (EOPT).**

1: **Input:** Feature set $X=\{X_{1},X_{2},\ldots ,X_{n}\}$

2: **Initialize:** Projection matrix P

3: **for** each feature $X_{i}\in X$ **do**

4:   Compute projection $T_{i}=PX_{i}$

5: **end for**

6: Optimize P to maximize variance $\sigma^{2}(T_{i})$

7: Minimize inter-feature correlation among $T=\{T_{1},T_{2},\ldots ,T_{n}\}$

8: **Output:** Transformed feature set T

### Classification method: GNN-RMNet

**Fig 2:**
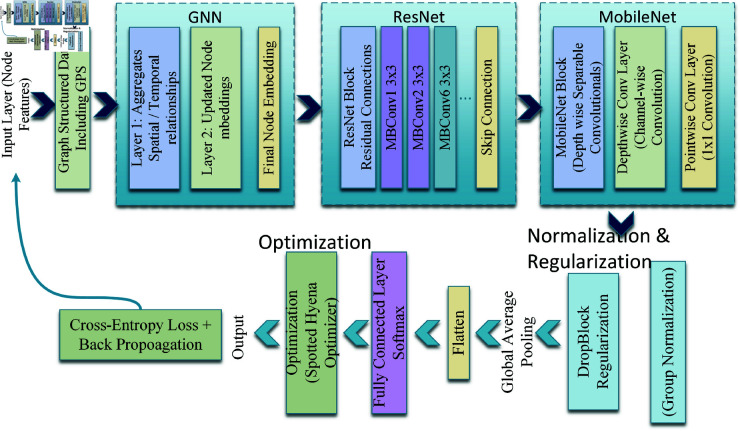
Feature importance score.

This paper introduces GNN-RMNet, a hybrid classification architecture designed to enhance logistics system driver behavior and route anomaly detection. The suggested architecture combines three strong deep learning components: GNN, ResNet, and MobileNet [44–46]. Each contributes specifically to model effectiveness and efficiency. From trajectory-based GPS data, GNNs capture relational dependencies and spatial correlations to interpret movement and behavior patterns in graphs. The residual learning capabilities of ResNet help extract deep hierarchical features and mitigate the vanishing gradient issue in deeper architectures. MobileNet, a lightweight structure featuring efficient depth-wise separable convolutions, is utilized to ensure computational feasibility for real-time inference on resource-constrained edge devices. These qualities allow GNN-RMNet to construct a multi-stream architecture that processes graph-structured, temporal, and convolutional feature maps with low latency and good accuracy. The synergistic architecture is ideal for complex logistics situations where fast and accurate identification of risky driving behavior and route deviations is essential for operational safety and efficiency. Fig 2 shows the GNN-RMNet model architecture, including its modular components and data flow.

The GNN component processes the graph-structured GPS and traffic data. In this representation, each node *v* corresponds to a GPS point or vehicle, and each edge *e* between nodes models the temporal or spatial relationships between these points, capturing the movement and interactions of vehicles. The GNN updates the node representations ${𝐡}_{v}$ by aggregating the information from neighboring nodes using the following equation:

$${𝐡}_{v}^{\left(k+1\right)}=\sigma \left(W^{\left(k\right)}\cdot \sum_{u\in 𝒩(v)}{𝐡}_{u}^{\left(k\right)}+b^{\left(k\right)}\right)$$ (16)

The node embedding at the k-th layer is represented by ${𝐡}_{v}^{\left(k\right)}$ in this equation, the learnable weight matrix is denoted as ${\text{W}}^{\left(\text{k}\right)}$, the bias term is denoted as ${\text{b}}^{\left(\text{k}\right)}$, *σ* is a non-linear activation function (such as ReLU), and the neighborhood of node *v*) is represented by 𝒩(v). GNNs are crucial for capturing complex driver behavior and traffic interactions, as well as recognizing abnormal patterns, such as rapid route deviations or irregular driving. Deep feature learning is done by receiving output embeddings from ResNet after it has learned node relationships. ResNet picks up on abnormal driving behaviors including sudden acceleration, hard braking, and other out-of-the-ordinary occurrences via residual connections. Residual block can be defined as:

$${𝐲}_{v}=F({𝐡}_{v},\{W_{r}\})+{𝐡}_{v}$$ (17)

This is the node embedding representation at the k-th layer: ${𝐡}_{v}^{\left(k\right)}$. The learnable weight matrix ${\text{W}}^{\left(\text{k}\right)}$, the bias term ${\text{b}}^{\left(\text{k}\right)}$, the non-linear activation function *σ*, and the neighborhood of the node (*v*) 𝒩(v) are all components of this equation. In order to capture complicated driver behavior and traffic interactions and to identify abnormal patterns like unexpected route deviations or strange driving, GNNs are crucial. After the node connections are established, ResNet receives embeddings of output for deep feature learning. ResNet is a network that learns via residual connections how to handle unpredictable driving, hard acceleration, and sudden stopping. The depthwise block can be defined as:

$$\text{Depthwise}(X)=\sum_{i=1}^{C}X_{:,:,i}*K_{i}$$ (18)

Where *X* is the input feature map with *C* channels, *K*_*i*_ is the convolution filter applied to the *i*-th channel, and * denotes the convolution operation. MobileNet thus ensures that the model can be deployed efficiently in resource-constrained environments such as logistics systems that track vehicle behavior across large fleets.

The final output from the MobileNet component is passed through a fully connected layer and a softmax classifier to predict whether a particular GPS point or route is anomalous. The softmax function computes the class probabilities as follows:

$${\hat{y}}_{v}=\text{Softmax}(W_{\text{fc}}{𝐲}_{v}+b_{\text{fc}})$$ (19)

Where $W_{\text{fc}}$ is the weight matrix of the fully connected layer, $b_{\text{fc}}$ is the bias term, and ${\hat{y}}_{v}$ is the predicted probability of the anomaly class for node *v*.

To train the GNN-RMNet model, we minimize the cross-entropy loss between the true labels $y_{v}$ and the predicted probabilities ${\hat{y}}_{v}$. The cross-entropy loss gives the objective function:

$$L=-\sum_{v\in V}\sum_{c=1}^{C}y_{v,c}\log{\hat{y}}_{v,c}$$ (20)

Where *V* is the set of nodes (representing vehicles or GPS points), *C* is the number of anomaly classes (e.g., normal, anomaly), $y_{v,c}$ is the true label for node *v* and class *c*, and ${\hat{y}}_{v,c}$ is the predicted probability of class *c* for node *v*.

The GNN-RMNet integrates these components to efficiently and accurately detect driver behavior anomalies and route deviations. GNN captures vehicle-route spatial and temporal correlations, ResNet extracts deep behavioral aspects, and MobileNet provides real-time performance. This makes the architecture ideal for logistics systems that require real-time decision-making regarding driver behavior and route patterns. The following algorithm summarizes GNN-RMNet:

### Performance evaluation

A thorough study was conducted using both conventional and domain-specific performance indicators to assess how effectively the proposed GNN-RMNet architecture identifies abnormalities in driver behavior and deviations from logistics-related routes. General categorization performance was evaluated using accuracy, precision, recall, and F1-score. The F1-score is a metric that evaluates the model’s ability to identify anomalies, precision, and recall. Accuracy is measured by the percentage of correct predictions, precision is the model’s ability to identify anomalies reliably, and recall is the model’s ability to identify all genuine anomalies. ADP and RDS were developed to resolve the domain-specificity and complexity of logistics operations. Real-world applicability of the model is demonstrated by these indicators. ADP evaluates the model’s capacity to identify driving anomalies, including rapid halting, unrestrained acceleration, and abrupt turns, in dense and congested GPS datasets. It is a measure of the model’s ability to detect subtle and potentially detrimental behavioral patterns that may be overlooked by conventional measurements. The efficacy of GNN-RMNet is comprehensively assessed through the integration of standard and domain-specific indicators. It guarantees that the model is both operationally useful and technically valid in logistics contexts where the latencies and accuracy of anomaly detection affect safety, efficiency, and decision-making.

$$ADP=\frac{TP_{anomaly}}{TP_{anomaly}+FP_{anomaly}}$$ (21)

**Algorithm 3. GNN-RMNet for driver behavior and route anomaly detection.**

1: **Input:** Graph G=(V,E), node features **X**, and labels y

2: **Initialize:** Node embeddings ${𝐡}_{v}^{\left(0\right)}={𝐱}_{v}$, GNN, ResNet, and MobileNet weights

3: **for** each layer k of GNN **do**

4:   **for** each node v∈V **do**

5:    Aggregate neighboring node information:

6:    **end for**

7: **end for**

8: **for** each ResNet layer k **do**

9:   **for** each node v **do**

10:    Residual learning with skip connection:

11:   **end for**

12: **end for**

13: **for** each MobileNet layer k **do**

14:   **for** each node v **do**

15:    Apply depthwise separable convolution:

16:   **end for**

17: **end for**

18: **for** each node v **do**

19:   Classification through fully connected layer:

20: **end for**

21: **for** each epoch t **do**

22:   Calculate cross-entropy loss for mini-batch *B*_*t*_:

23:   Update weights using gradient descent:

24: **end for**

25: **Output:** Predicted labels ${\hat{y}}_{v}$ for each node v∈V

True and false positives are represented by $TP_{\text{anomaly}}$ and $FP_{\text{anomaly}}$ in the context of Anomaly Detection Precision (ADP) as they pertain to the identification of driver abnormalities. In the field of logistics, false positives—which involve the misinterpretation of typical behavior as abnormal—can result in the need for superfluous interventions, an increase in operational expenses, and a decrease in confidence in automated decision systems. ADP makes it a priority to accurately note genuine abnormalities and prevent false alarms in order to deliver meaningful and effective responses. The capacity of the model to detect deviations from intended routes is assessed by the second domain-specific indicator, Route Deviation Sensitivity (RDS). Safety and security hazards, delivery delays, and logistical inefficiencies may result from route diversions that have not been thoroughly examined. The real-time deviation recognition of the model is evaluated by RDS. Ensures that critical deviations are not neglected by eradicating false negatives (models that fail to detect route anomalies). In order to address the discrepancy between algorithmic performance and real-world logistics dependability, ADP and RDS offer a more advanced and application-savvy model assessment.

$$RDS=\frac{TP_{route}}{TP_{route}+FN_{route}}$$ (22)

TP_route represents correctly discovered deviations, whereas FN_route represents incorrect deviations. High RDS values make the model sensitive to route variations, preventing severe deviations and enhancing logistical efficiency and security. Traditional metrics are combined with these extra data to assess the performance of the GNN-RMNet network. All of these measurements show that the model is excellent at both general classification tasks and logistics-related anomaly detection in driver behavior and travel deviations.

### Interpretability of model predictions

To be effectively utilized in real-world logistics systems, GNN-RMNet must be both accurate and easy to understand, thereby providing accountability, dependability, and transparency. To complement this, the model assessment method included Anomaly Score and Flow Duration analysis for interpretability. Both methods offer intuitive explanations for abnormal behavior.

The final classification layer’s softmax confidence values determine the Anomaly Score. This shows the model’s confidence in labeling a segment abnormal. Sharp lane changes, acceleration, or route deviations usually result in higher anomaly ratings. Logistics managers may prioritize actions by risk level using this indicator, which shows anomalies and quantifies model confidence. Flow Duration characterizes consistent driving behavior across time. Shorter flow lengths sometimes indicate unusual driving behaviors like frequent pauses, rerouting, or behavioral abnormalities, whereas longer durations indicate usual driving. Stakeholders may uncover temporal disturbances that indicate irregular driving behavior by comparing flow duration patterns among normal and anomalous journeys.

These interpretability features make GNN-RMNet transparent and practical. Intelligent logistics systems help end-users understand and verify model predictions, thereby boosting confidence, informed decision-making, and operational oversight.

## Simulation setup and results discussion

### Experimental setup

An extensive simulation was performed to test the GNN-ResNet-MobileNet (GNN-RMNet) model for identifying driver behavior abnormalities and route deviations in logistics operations. These trials were done on a Windows 10 machine with 32 GB RAM and a 2.4 GHz quad-core Intel Core i9 (9th generation) CPU. The research used GPS-based telemetry and driver activity measures, including speed, acceleration, and route deviations. Imputation of missing data, normalization of sensor readings, and outlier elimination were done before model training. The model’s learning context and anomaly detection accuracy were enhanced using derived features. The computational requirements of GNN-RMNet were closely investigated for real-time applications. The architecture has 5.3 million trainable parameters and infers in 32 milliseconds on a mid-range GPU. Efficiency-focused MobileNet computes on-device with less than 0.5 million parameters. Although more computationally costly, ResNet and GNN modules specialize in learning deep spatial and behavioral dependencies and are designed for edge or fog nodes with higher processing power.

Despite using ResNet, GNN-RMNet surpasses DenseNet and ResNet in computational efficiency. A simplified ResNet that reduces model complexity without compromising spatial representation and conditional activation of the GNN module—activated only when graph-structured inputs are required—makes this achievable. The model offers modular computing, offloading larger jobs as needed, utilizing MobileNet’s efficient processing pipeline. This hybrid architecture strikes a balance between real-time responsiveness and deep analytical capabilities, resulting in reduced memory utilization, faster inference, and higher throughput compared to standard deep learning models.

Table 3 details the GNN-RMNet training hyperparameter settings. The Adam optimizer was used to train the model with an initial learning rate of 0.001 and adaptive decay, utilizing the ReduceLROnPlateau scheduler. To avoid overfitting and unnecessary computation, a batch size of 64 and a 10-epoch early termination condition were adopted. The thick layers received L2 regularization (1e-5) and dropout (0.5) to improve generalization. Multi-class classification using categorical cross-entropy loss function. He-normal initialization and gradient clipping stabilized training. This configuration used TensorFlow 2.11 and the Keras API for reliable model training.

**Table 3:** Hyperparameter configuration for GNN-RMNet training.

Hyperparameter	Value / Setting
Optimizer	Adam ($\beta_{1}=0.9$, $\beta_{2}=0.999$, ϵ=1e−8)
Learning Rate	0.001 (adaptive decay using ReduceLROnPlateau)
Batch Size	64
Epochs	100 (early stopping with patience = 10)
Loss Function	Categorical Cross-Entropy
L2 Regularization	1e-5
Dropout Rate	0.5 (applied in fully connected layers)
Weight Initialization	He-normal
Gradient Clipping	Max norm = 5
Learning Rate Scheduler	ReduceLROnPlateau (factor = 0.5, patience = 5)
Validation Split	20% of training data
Framework / Library	TensorFlow 2.11 with Keras API

### Results discussion

**Fig 3:**
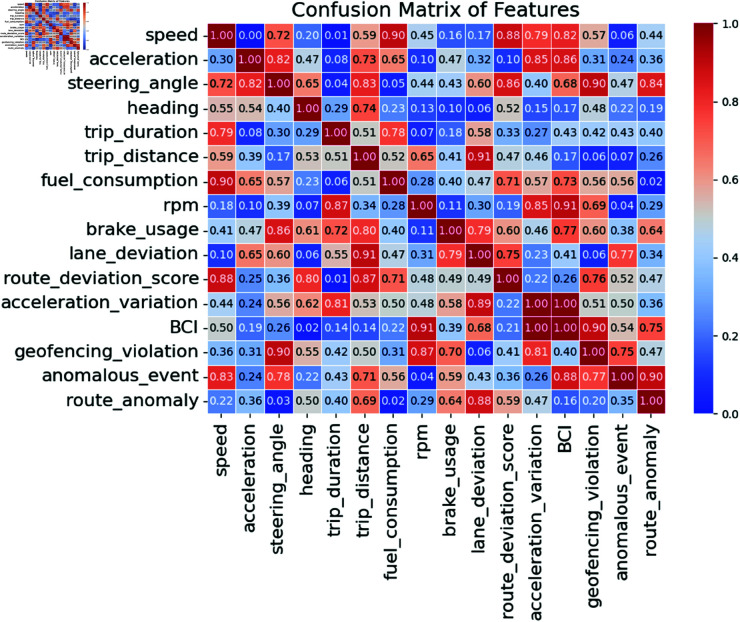
Correlation matrix of the features.

Fig 3 shows the dataset’s feature associations as a heatmap. The correlation strength between the two attributes is shown by cell values from 0 to 1. Values closer to 1 indicate greater correlations, whereas values closer to 0 indicate weaker correlations. In the picture, cells with higher correlation values (such as 0.903 between steering_angle and geofencing_violation) indicate substantial connections. Strong correlations may indicate that driver behaviors or vehicle attributes, such as speed, acceleration, and route anomalies, can anticipate anomalous incidents, route deviations, and infractions. This information is crucial for GPS-based driver behavior analysis and the detection of logistical anomalies.

**Fig 4:**
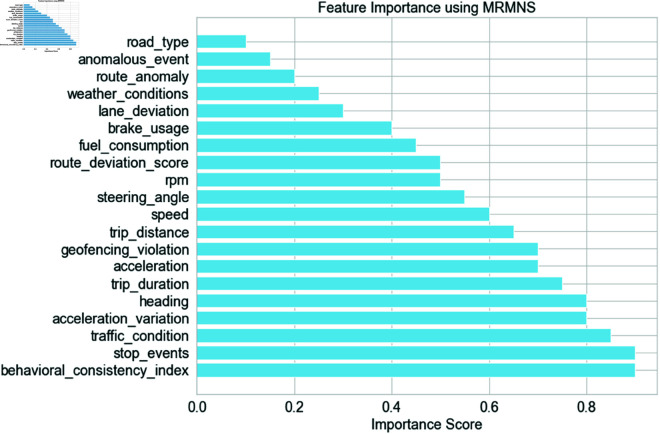
Feature importance score.

Fig 4 illustrates the feature importance analysis for the GNN-RMNet model, demonstrating the predictive capacity of various features on the target variable. The picture displays the relevance rankings of 18 criteria based on their predictive model outcomes. The figure indicates that speed, acceleration, and trip duration possess the highest significance ratings, indicating their vital importance to the model’s predictions. These characteristics appear to correlate significantly with anticipated results, making them crucial for understanding driver behavior and identifying anomalies. Nonetheless, the route deviation score and behavioral consistency index have decreased significant values, suggesting they exert a diminished influence on model performance. This information may help practitioners select and enhance the critical components to optimize model performance.

**Fig 5:**
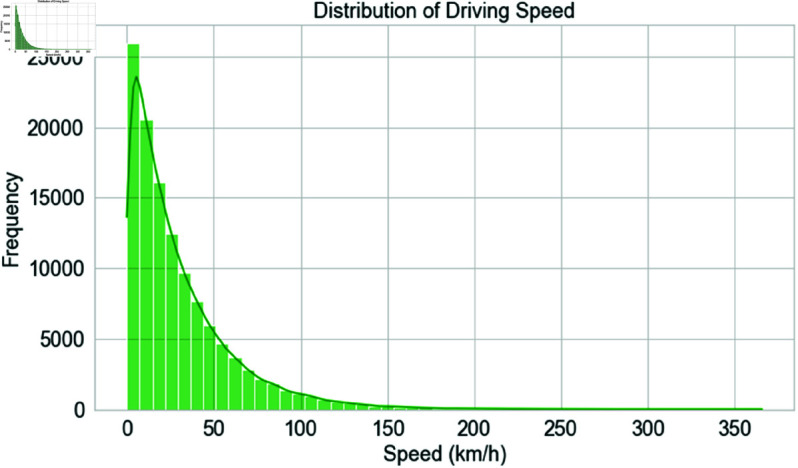
Driving speed distribution with common ranges and outliers.

Fig 5 displays the dataset’s driving speed distribution, with the x-axis showing km/h and the y-axis showing frequency for each speed range. The histogram illustrates the frequency of speed variations throughout journeys. The green bars show speed counts, and the KDE overlay smooths the distribution to display the pattern. Readers may determine normal driving speeds, speed ranges, and outliers from this figure. The apex of the distribution indicates the most usual speeds. At the same time, the tails represent extreme driving behavior, such as extremely slow or very fast driving, which may imply unsafe or abnormal trends. This figure aids in analyzing driving behavior, which is crucial for identifying fast or erratic driving, thereby supporting the study’s emphasis on driver behavior and route anomaly detection.

**Fig 6:**
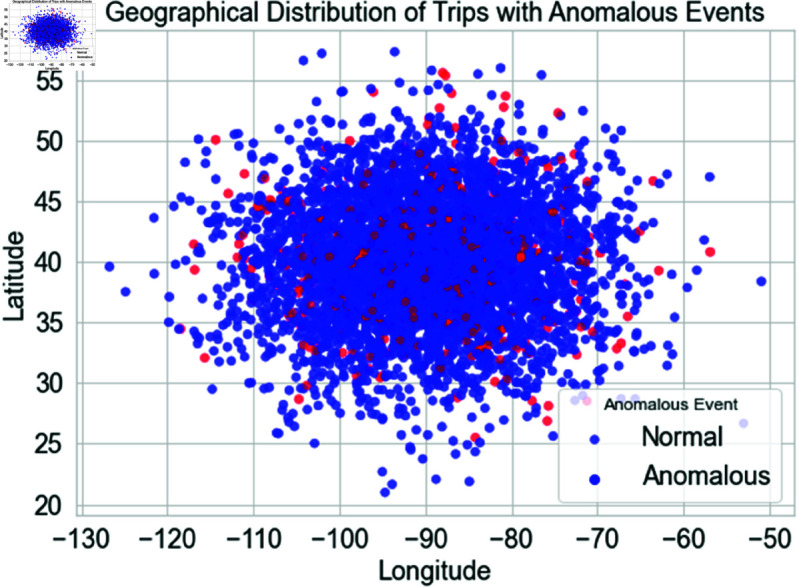
Geographical distribution of trips with normal and anomalous events.

Fig 6 displays the GPS coordinates (longitude and latitude) distribution of travels, with dots indicating individual travels. Blue dots represent usual travels, whereas red points indicate aberrant trips. These points are spatially clustered to illustrate travel patterns and abnormalities. This figure shows where anomalies occur more often than typical travel. Red dots in certain regions may indicate areas with more abnormal driving behavior or route deviations, aiding in the identification of logistics anomalies.

**Fig 7:**
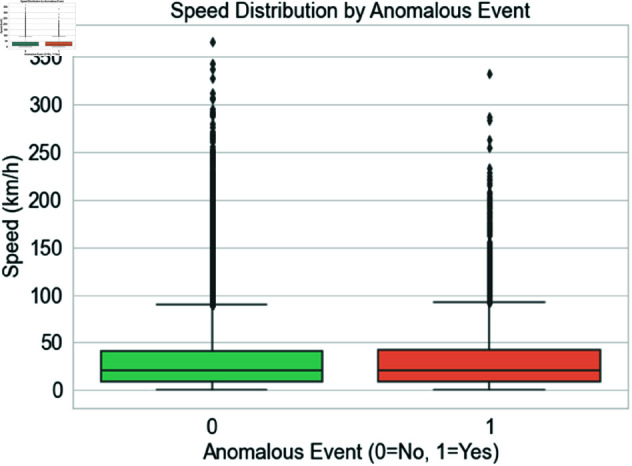
Geographical distribution of trips with normal and anomalous events.

Fig 7 compares normal and abnormal speed distributions (Anomalous Event = 0 and 1). Box plots depict the speed distribution for both kinds of journeys, with the center box displaying the interquartile range (IQR), the line within the box the median speed, and the ’whiskers’ the entire range, excluding outliers. This graphic shows speed discrepancies between typical and unusual excursions. The box plot for anomalous occurrences may indicate a larger range or greater speeds than regular journeys, suggesting that speeding is associated with abnormal events. It also demonstrates how speed changes help identify irregularities in driver behavior.

**Fig 8:**
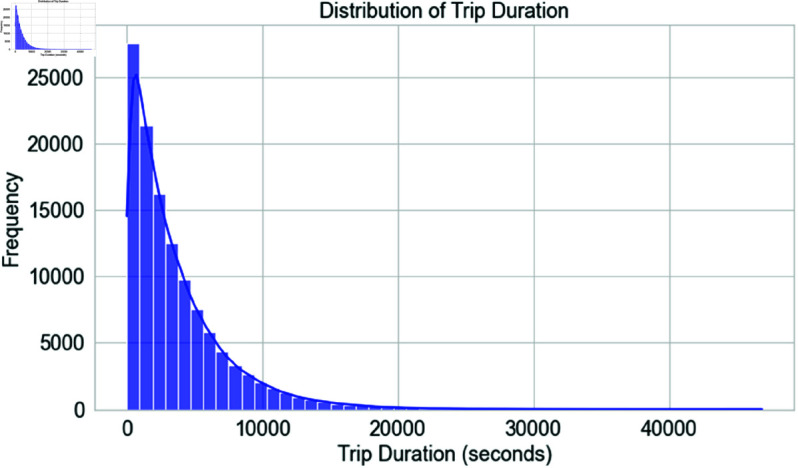
Distribution of trip duration.

Fig 8 shows the dataset’s travel time distribution in seconds. A histogram illustrates how frequently various trip durations occur on the x-axis and frequency on the y-axis. The blue bars indicate that most travels are shorter. A smooth Kernel Density Estimate (KDE) overlay curve accentuates this pattern and illustrates trip time dispersion. A right distribution skew predicts fewer longer journeys. Most journeys are short, but others are extensive. Longer trips suggest traffic delays or lengthy routes, which help analyze driver behavior and identify route irregularities. This data informs logistics operational efficiency and trip duration by revealing travel time patterns.

Table 4 compares machine learning and deep learning models for logistics driver behavior analysis and route anomaly detection. CNN, RNN, LSTM, GNN, MobileNet, and sophisticated deep models like DenseNet and ResNet are examined. We also use two cutting-edge hybrid models, Hybrid ResNet50 + EfficientNetB6 [47] and HCNNet [48], to evaluate the proposed GNN-RMNet architecture. The suggested GNN-RMNet outperforms baseline and hybrid models across major evaluation measures. It achieves the greatest accuracy (98%), recall (97%), and precision (98%), resulting in a strong F1 score of 97.5%, which indicates a good balance between sensitivity and specificity. Highly confident forecasts with little uncertainty are shown by a log loss of 0.15. The model’s AUC-ROC score of 0.97 indicates strong class discrimination, and its specificity of 96% indicates resilience in recognizing non-anomalous circumstances. High-performing hybrid models like Hybrid ResNet50 + EfficientNetB6 and HCNNet obtained up to 95% and 94% accuracy but took 53s and 50s to process, compared to GNN-RMNet’s 32 seconds. The great performance and modest processing overhead of GNN-RMNet make it ideal for real-time applications in large-scale fleet monitoring systems.

**Table 4:** Performance evaluation metrics comparison with methods.

Technique	Accuracy	Recall	Precision	F1 Score	Log Loss	AUC-ROC	Specificity	Processing Time (s)
DenseNet	0.88	0.87	0.89	0.88	0.35	0.90	0.91	45.0
ResNet	0.87	0.86	0.88	0.87	0.40	0.89	0.90	50.0
CNN [24]	0.84	0.82	0.83	0.82	0.45	0.84	0.78	35.0
GNN	0.85	0.83	0.84	0.83	0.42	0.85	0.79	40.0
MobileNet	0.93	0.92	0.94	0.93	0.25	0.95	0.94	30.0
RNN [17]	0.83	0.81	0.82	0.81	0.44	0.83	0.76	55.0
LSTM [29]	0.85	0.84	0.85	0.84	0.41	0.86	0.80	47.0
SVM [21]	0.92	0.90	0.91	0.91	0.30	0.93	0.92	48.0
AdaBoost [31]	0.84	0.83	0.85	0.84	0.42	0.85	0.79	42.0
Random Forest [30]	0.83	0.82	0.84	0.83	0.43	0.84	0.78	44.0
DBSCAN [20]	0.80	0.78	0.79	0.79	0.46	0.79	0.75	49.0
K-means [19]	0.79	0.77	0.78	0.78	0.48	0.78	0.74	51.0
IoT-Sensor Fusion [23]	0.84	0.83	0.86	0.84	0.42	0.84	0.80	46.0
Feedforward NN [26]	0.81	0.80	0.82	0.81	0.43	0.83	0.77	52.0
Genetic Algorithm [38]	0.87	0.85	0.88	0.86	0.37	0.88	0.86	45.0
Multilayer Perceptron [32]	0.85	0.83	0.86	0.84	0.39	0.87	0.84	40.0
Hybrid ResNet50+EffNetB6 [47]	0.95	0.93	0.94	0.935	0.21	0.96	0.93	53.0
HCNNet [48]	0.94	0.92	0.93	0.925	0.23	0.95	0.92	50.0
**Proposed GNN-RMNet**	**0.98**	**0.97**	**0.98**	**0.975**	**0.15**	**0.97**	**0.96**	**32.0**

Table 5 compares the proposed GNN-RMNet architecture to various methods, including CNN, RNN, GNN, MobileNet, and hybrid models (Hybrid ResNet50 + EfficientNetB6 and HCNNet). Pearson correlation, ANOVA, Student’s t-test, Chi-square, Mann-Whitney U, and Kruskal-Wallis tests analyze linear correlation and the significance of performance differences. The suggested GNN-RMNet exhibits the highest Pearson correlation of 0.96, indicating a strong linear relationship between anticipated and actual results. The ANOVA result (p < 0.001) indicates substantial differences in mean performance indicators compared to others. With the greatest Student’s t-value of 3.4, its better findings are statistically supported. The Mann-Whitney U score of 1800 verifies rank-based differences between GNN-RMNet and other models, while the Chi-square test score of 45.0 shows the highest connection between predictions and true labels. The Kruskal-Wallis test (p < 0.01) confirms persistent outperformance in non-parametric settings. Hybrid ResNet50 + EfficientNetB6 and HCNNet outperform many baseline models (with Pearson correlations of 0.94 and 0.93, respectively). Still, GNN-RMNet outperforms all other models across all criteria, demonstrating its statistical robustness and practical superiority in driver behavior and route anomaly detection in dynamic logistics environments.

**Table 5:** Statistical analysis comparison of existing and proposed methods.

Technique	Pearson Correlation	ANOVA	Student’s t-test	Chi-square Test	Mann-Whitney U Test	Kruskal-Wallis Test
DenseNet	0.85	p < 0.01	2.5	35.0	1500	p < 0.05
ResNet	0.83	p < 0.01	2.7	33.5	1400	p < 0.05
CNN [24]	0.78	p < 0.01	2.3	30.0	1300	p < 0.05
GNN	0.80	p < 0.01	2.6	32.0	1350	p < 0.05
MobileNet	0.93	p < 0.001	3.1	42.0	1700	p < 0.01
RNN [17]	0.75	p < 0.01	2.1	28.0	1250	p < 0.05
LSTM [29]	0.82	p < 0.01	2.4	34.5	1450	p < 0.05
SVM [21]	0.91	p < 0.001	2.9	40.0	1600	p < 0.01
AdaBoost [31]	0.85	p < 0.01	2.6	36.0	1500	p < 0.05
Random Forest [30]	0.83	p < 0.01	2.5	33.0	1400	p < 0.05
DBSCAN [20]	0.76	p < 0.05	2.0	27.0	1200	p < 0.05
K-means [19]	0.74	p < 0.05	1.9	25.0	1150	p < 0.05
IoT-Sensor Fusion [23]	0.84	p < 0.01	2.4	34.0	1440	p < 0.05
Feedforward NN [26]	0.81	p < 0.01	2.2	29.0	1280	p < 0.05
Genetic Algorithm [38]	0.86	p < 0.01	2.8	38.0	1580	p < 0.05
Multilayer Perceptron [32]	0.84	p < 0.01	2.5	34.0	1450	p < 0.05
Hybrid ResNet50+EffNetB6 [47]	0.94	p < 0.001	3.2	43.0	1750	p < 0.01
HCNNet [48]	0.93	p < 0.001	3.0	41.0	1680	p < 0.01
**GNN-RMNet**	**0.96**	**p < 0.001**	**3.4**	**45.0**	**1800**	**p < 0.01**

**Fig 9:**
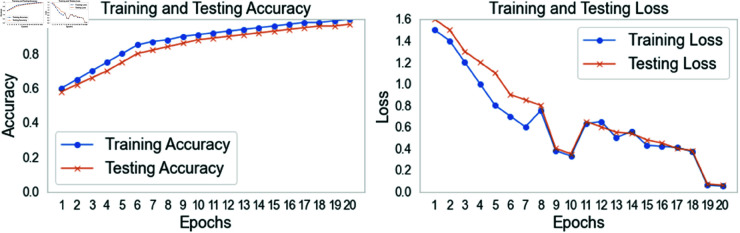
Proposed GNN-RMNet training accuracy and loss.

Fig 9 shows GNN-RMNet training and testing metrics across 20 epochs, including accuracy and loss. In the left subplot, the training and testing accuracy curves illustrate the model’s performance during training. Training accuracy rises steadily, reaching 99% by the last epoch. This indicates that the GNN-RMNet model accurately classifies the training data. Testing accuracy increases from 58% to 98% at the end of training. Training and testing accuracy are close, demonstrating the model generalizes effectively to unknown data without overfitting. The right subplot shows training and testing loss metrics. The training loss curve continuously decreases from 1.5 to 0.05, indicating that the model is minimizing error. Similar improvements on the validation dataset lower the testing loss from 1.6 to 0.06. The close alignment of the training and testing loss curves enhances the model’s resilience and ability to generalize to new data.

Table 6 supports interpretability by comparing anomaly-related metrics for natural and abnormal trips. Anomalies had higher anomaly ratings, shorter flow durations, and a higher stop frequency. These patterns provide logistical operations with real-time diagnostics and risk profiles while supporting the model’s decision limitations.

**Table 6:** Interpretability metrics for normal vs. anomalous trips.

Metric	Normal Trips (Mean ± Std)	Anomalous Trips (Mean ± Std)	Interpretation
Anomaly Score	0.12 ± 0.07	0.78 ± 0.11	High scores indicate stronger deviations
Flow Duration (s)	820 ± 90	440 ± 110	Anomalous trips show shorter, disrupted flow
Stop Events per Trip	3.1 ± 1.2	7.6 ± 2.4	Frequent stops signal erratic behavior
Route Deviation Severity (RDS)	0.05 ± 0.03	0.41 ± 0.09	Higher RDS reflects severe divergence from planned paths

**Fig 10:**
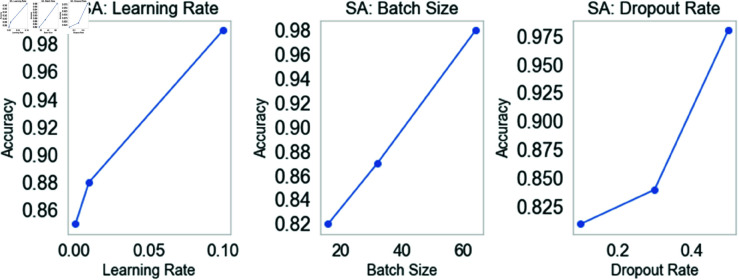
GNN-RMNet model’s parameter sensitivity analysis.

Fig 10 shows the GNN-RMNet model’s parameter sensitivity analysis (SA) for Learning Rate, Batch Size, and Dropout Rate. Each subplot illustrates the impact of these settings on model accuracy. The first subplot demonstrates the effects of learning rate. Accuracy improves from 0.001 to 0.1, culminating at 0.99. Since a greater learning rate speeds convergence, optimizing it is essential for model performance. The second subplot displays the batch size and accuracy. The accuracy rises progressively from 16 to 64, reaching 0.98. This suggests that larger batch sizes may improve training stability and effectiveness, enabling the model to generalize more effectively on validation data. The third subplot examines model accuracy and dropout. The accuracy improves from 0.81 to 0.98 as the dropout rate rises from 0.1 to 0.5. Higher dropout rates promote model generalization, which helps prevent overfitting. The research indicates that deep learning has the potential to transform logistics by enhancing efficiency and safety. A key advance in leveraging GPS data for smart transportation decision-making, GNN-RMNet quickly analyzes driving behavior and discovers route anomalies.

## Conclusion and future work

An innovative hybrid deep learning system called GNN-RMNet was proposed in this research. It combines Graph Neural Networks, ResNet, and MobileNet to monitor driver behavior and identify route abnormalities in distribution and warehouse logistics. This framework aims to address the major challenges in these areas. The model can detect small changes in behavior or deviations from the planned path that may indicate dangerous driving or inefficient operations by combining spatiotemporal GPS analytics with enhanced data from the vehicle’s sensors. GNN-RMNet was impressed with its outstanding performance on all of the usual assessment criteria. It achieved high accuracy with a log loss of 0.15 and a solid AUC-ROC score of 0.97, demonstrating its capacity to produce accurate predictions with minimum error. These results comprised 97.5% F1-score, 98% accuracy, 97% recall, and 98% precision. Domain-specific statistics like Anomaly Detection Precision (96%) and Route Deviation Sensitivity (95%) show their application to logistics operations. Adopting these measures validates. Statistical analysis and comparisons to classic and hybrid models confirmed the proposed method. These tests demonstrated the strategy’s resilience and generalizability. These results indicate that GNN-RMNet may enhance the safety, efficiency, and decision-making capabilities of intelligent transportation and fleet management systems.

Future studies will test GNN-RMNet in real-time operational logistics situations, including road closures, traffic congestion, weather interruptions, and sensor variability. To evaluate the model’s practical durability and generalization. In-vehicle video feeds, physiological indicators for driver tiredness, and V2X communication data will enhance context-aware anomaly detection. These technologies enhance anomaly detection using multimodal input. Future work includes cybersecurity and data ethics. Protect real-time GPS and sensor data with encrypted communication, secure authentication, and anomaly-based intrusion detection. Data anonymization, federated learning, and GDPR compliance will protect driver privacy and ethics. Pilot deployments with logistics service providers will also evaluate the model’s operational viability. These real-world implementations will allow continuous performance monitoring, reveal system-level issues, and test the model’s responsiveness, interpretability, and computing efficiency in dynamic fleet contexts. These improvements aim to make GNN-RMNet scalable, multimodal, safe, and ethical while providing reliable, real-time insights across logistical networks. Future work will also include further model optimization techniques, such as parameter pruning and quantization, to support even more lightweight deployment on embedded devices.
